# Chronic Alcohol Exposure of Cells Using Controlled Alcohol-Releasing Capillaries

**DOI:** 10.3390/cells10051120

**Published:** 2021-05-06

**Authors:** Wanil Kim, Hye-Seon Jeong, Sang-Chan Kim, Chang-Hyung Choi, Kyung-Ha Lee

**Affiliations:** 1 Division of Cosmetic Science and Technology, Daegu Haany University, Hanuidae-ro 1, Gyeongsan-si 38610, Gyeongsangbuk-do, Korea; wkim@gnu.ac.kr (W.K.); djs4736@dhu.ac.kr (H.-S.J.); 2 Department of Biochemistry and Institute of Health Sciences, School of Medicine, Gyeongsang National University, Jinju 52727, Korea; 3 College of Korean Medicine, Daegu Haany University, Hanuidae-ro 1, Gyeongsan-si 38610, Gyeongsangbuk-do, Korea; sckim@dhu.ac.kr

**Keywords:** alcohol, chronic, liver, capillary

## Abstract

Alcohol is one of the main causes of liver diseases such as fatty liver, alcoholic hepatitis, and chronic hepatitis with liver fibrosis or cirrhosis. To reproduce the conditions of alcohol-induced liver diseases and to identify the disease-causing mechanisms at the cellular level, several methods have been used to expose the cells to ethanol. As ethanol evaporates easily, it is difficult to mimic chronic alcohol exposure conditions at the cellular level. In this study, we developed a glass capillary system containing ethanol, which could steadily release ethanol from the polyethylene tubing and hydrogel portion at both sides of the capillary. The ethanol-containing capillary could release ethanol in the cell culture medium for up to 144 h, and the concentration of ethanol in the cell culture medium could be adjusted by controlling the number of capillaries. A long-term exposure to ethanol by the capillary system led to an increased toxicity of cells and altered the cellular physiologies, such as increasing the lipid accumulation and hepatic transaminase release in cells, as compared to the traditional direct ethanol addition method. Ethanol capillaries showed different gene expression patterns of lipid accumulation- or chronic alcoholism-related genes. Our results suggest that our ethanol-containing capillary system can be used as a valuable tool for studying the mechanism of chronic alcohol-mediated hepatic diseases at the cellular level.

## 1. Introduction

Alcohol consumption can have beneficial social and psychological effects. However, habitual abuse of alcohol causes various mental and physical illnesses in individuals. In 2019, 25.8% of the adults in the United States participated in binge drinking, and during the past month, 6.3% of the adults were involved in heavy drinking [1]. The number of annual alcohol-related deaths in the United States was 95,000, of which 68,000 were males and 27,000 were females [2,3]. Thus, alcohol abuse leads to significant social and economic losses, and acute and chronic alcohol dependence causes serious health problems. According to the International Classification of Disease-10, alcohol abuse causes 25 diseases, majorly diabetes, cancer, neuropsychiatric conditions, and cardiovascular diseases [4].

The liver performs many critical functions in the body, and liver diseases, also referred to as hepatic diseases, can cause significant damage to the body. A variety of causes, such as parasitic and viral infections, hereditary conditions with genetic disorders, and liver toxins such as alcohol, can cause hepatic diseases [5,6,7,8,9,10]. Among the various causes of hepatic diseases, alcoholic injury affects the individuals and their families the most, although alcohol-related harm can be prevented [10,11]. In addition to alcohol being the major cause of liver diseases in Western countries, the liver is the main site for alcohol metabolism [12]. As the liver is a major target organ for alcohol-mediated injury, alcohol causes fatty liver disease, alcoholic hepatitis, chronic hepatitis, liver fibrosis, and cirrhosis [13,14,15,16]. In particular, the risk of developing cancer is higher in hepatitis B and C patients than in non-drinkers, even with a small amount of alcohol [17,18]. Many clinical, animal-based, cellular, and molecular-level studies have been conducted to understand the mechanisms and signaling pathways of alcohol-induced liver diseases [12,16,19,20]. Recent studies on alcoholic liver diseases have linked genetic predispositions due to *PNPLA3*, *ALDH2*, *CYP2E1*, and *NFE2L2* to the status [21,22,23,24]. However, to date, it is difficult to predict and treat liver diseases due to chronic alcohol intake.

To understand the cellular and molecular mechanisms of acute alcohol-mediated liver disease, a direct administration of alcohol into the cell culture medium is commonly used to mimic acute alcohol use in organisms [25]. Since ethanol evaporation from the culture medium is one of the significant causes for misunderstanding the response of a long-term exposure of cells to alcohol, chronic alcohol exposure conditions are difficult to mimic at the cellular level [26]. To overcome this limitation, several methods, such as sealing the culture plate to reduce evaporation, periodic addition of ethanol into the culture medium, and saturating the ethanol concentration in the air of the incubator, have been performed [27,28,29]. The most commonly used method is to supply ethanol while changing the medium at regular intervals, for example at 24 h [30]. However, this method leads to a change in alcohol concentration during cultivation with a probable contamination of the medium, which leads to a misunderstanding of the results by dampening the reliability of the study. Saturating the chamber atmosphere over the culture medium with ethanol or sealing the culture dishes with Parafilm^®^ can be an alternative method to keep the cells exposed to ethanol [26]. However, these methods can affect cell viabilities by interfering with the proper exchange of oxygen and carbon dioxide during prolonged (>24 h) cultivation. Therefore, the establishment of a simple and appropriate method to analyze the physiological responses of hepatocytes to chronic alcohol exposure is difficult, and it is very important to establish a method for the sustained and controlled release of ethanol over a long period of time, i.e., for several days.

In this study, we attempted to develop an easy apparatus to overcome the limitation of ethanol evaporation on prolonged exposure. We devised a simple carrier that could constantly release ethanol, and we checked whether the ethanol concentration in the culture medium remained constant for several days while incubating it together by simply placing it in a culture dish. This method is simple, and alcohol concentration in the culture medium can be easily controlled because you only need to float the small capillary structure in the culture medium. This method can block the possibility of changes in ethanol concentration and cross-contamination.

## 2. Materials and Methods

### 2.1. Fabrication of Glass Capillary Device

A glass capillary with a circular cross-section (1 mm outer diameter) was used as the container for ethanol. To sterilize the capillaries, they were put into an aqueous solution of 70% ethanol and subsequently exposed to ultraviolet irradiation (254 nm) for 1 h. To enclose ethanol within the capillary, we used a polyethylene (PE) tubing stopper (950 μm inner diameter), partially filled with polyethylene glycol diacrylate (PEG-DA, M_n_ = 700), which was transformed into a PEG hydrogel (estimated pore size: 1 nm) upon exposure to ultraviolet irradiation.

### 2.2. Cell Culture and Ethanol Treatment

HepG2 cells (Korean Cell Line Bank) were cultured in minimal essential medium (HyClone, Chicago, IL, USA) supplemented with 10% fetal bovine serum (FBS, HyClone) and 1% antibiotics (HyClone). L-02 cells were cultured in Dulbecco’s modified Eagle’s medium (Hyclone) supplemented with 10% FBS and 1% antibiotics. Cells were maintained in a humidified incubator with 95% ambient air and 5% CO_2_ at 37 °C.

For ethanol treatment using the direct addition, cells were seeded in 96-well plates or 6-well plates, and they were incubated for 24 h. Subsequently, the indicated concentration of ethanol (Sigma-Aldrich, Saint Louis, MO, USA) was directly added to the culture medium, and the mixture was incubated for the indicated time period. In the case of capillary system, four capillaries, providing an ethanol concentration of up to 86 mM (0.5% v/v), or 10 capillaries, providing an ethanol concentration of up to 171 mM (1% v/v), were used per well of a 6-well plate. The capillaries were carefully placed into the culture medium, and the plates were incubated for the indicated periods of time. The culture dishes were not wrapped with Parafilm^®^ or another film and were handled as per the standard mammalian cell culture method. All the cultivation procedures and cell culture wares were identical for both methods, except for the type of the ethanol exposure methods employed. The ethanol-containing capillaries were always floating on top of the culture medium.

### 2.3. RNA Quantification

Total RNA was extracted from HepG2 cells using TRIzol (Invitrogen, Waltham, MA, USA). RNA was reverse transcribed using GoScript^TM^ Reverse Transcription Mix, Oligo(dT) (Promega, Madison, WI, USA), according to manufacturer’s instructions. mRNA expression was quantified using quantitative real-time PCR using the QuantStudio 3 real-time PCR system (Applied Biosystems) with TaqMan Universal PCR Master Mix (Thermo Fisher Scientific, Waltham, MA, USA) or SYBR Green PCR Master Mix (Thermo Fisher Scientific, Waltham, MA, USA). Probes for each gene were selected from the Universal Probe Library System (Roche, Basel, Switzerland) using the ProbeFinder Assay Design Software. The primer sequences and probe numbers for each gene are described below. 

*ACTG1*—ttttggttttctactgttatgtgagaa/tttttggttacggcagcact (#57);

*TLR3*—agagttgtcatcgaatcaaattaaag/aatcttccaattgcgtgaaaa (#80);

*UROC1*—ttgccttgggggttacaat/ggggaccgatgtagcagtag (#62);

*CSAG1*—agatgtccaggaaaccacga/gggaacctctttggtgttga (#19);

*CSAG3*—aatgcgctgtgagtttctagc/cagaccagcgtctctcaaca (#54);

*RPL32*—gctgccatctgttttacgg/tgactggtgcctgatgaact (#46);

*FASN*—acagggacaacctggagttct/ctgtggtcccacttgatgagt;

*SREBP1C*—gacaggtgaagtcggcgc/catgtcttcgaaagtgcaatcc;

*TNFα*—TGCTTGTTCCTCAGCCTCTT/ATGGGCTACAGGCTTGTCACT;

*MCP1*—CCCCAGTCACCTGCTGTTAT/TGGAATCCTGAACCCACTTC.

### 2.4. Cell Viability and Ethanol Measurement

Cell viability was measured using the Cell Counting Kit-8 (Dojindo, Kumamoto, Japan) or the Countess II automated cell counter (Thermo Fisher Scientific), according to manufacturer’s instructions.

Ethanol containing phosphate-buffered saline (PBS) or culture medium was obtained at the indicated time points, and the concentration of ethanol was measured using the Ethanol Assay Kit (BioAssay Systems, Hayward, CA, USA) with an Infinite M nano microplate reader (Tecan, Zurich, Switzerland), according to manufacturer’s instructions. 

### 2.5. Oil Red O Staining

To visualize lipid accumulation in HepG2 cells, Oil Red O staining was performed according to a previously described method [31]. Briefly, cells were fixed in 4% formaldehyde solution at room temperature for 15 min, and they were washed with 60% isopropanol. The cells or slides were immersed in freshly prepared Oil Red O working solution (Sigma-Aldrich) for 15 min, and subsequently, they were washed with distilled water four times. Finally, the cells were analyzed using a light microscope, and they were photographed using a Leica camera. To measure Oil Red O staining, 100% isopropanol was added to each well, and the plate was incubated with shaking at room temperature for 10 min to elute the incorporated Oil Red O. Samples (200 μL) were quantified using spectrophotometry at 520 nm. 

### 2.6. Statistical Analysis

Statistical parameters, including the definitions and exact values of n (number of biological repeats), distributions, and deviations, are reported in the figures and their corresponding legends. All quantitative data are presented as mean ± standard error of the mean. Comparisons between two groups were analyzed using two-tailed unpaired Student’s *t*-tests. For comparisons between more than two groups, one-way analysis of variance was used with Tukey’s post hoc test. A two-way analysis of variance was used with Tukey’s post hoc test to estimate the mean differences between groups that had been split into two independent variables. Statistical significance was set at *p* < 0.05. Statistical analyses were performed using GraphPad Prism.

## 3. Results

### 3.1. The Glass Capillary Device Led to a Controlled Release of Ethanol 

**Figure 1:**
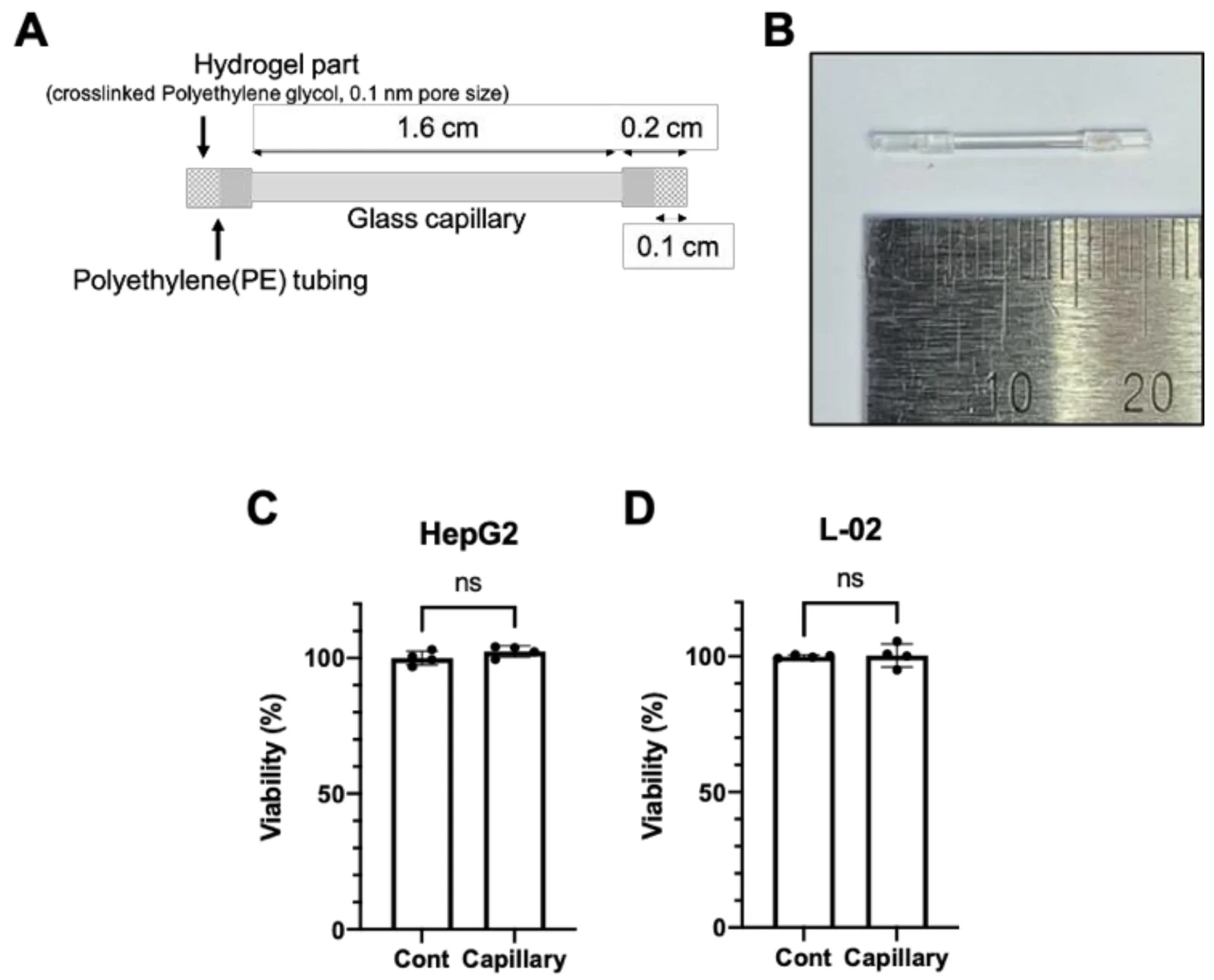
Glass capillary device. (**A**) Schematic diagram of the device containing a glass capillary tube connected to a polyethylene (PE) tubing (0.2 cm) on both sides. Half of the PE tubing was filled with polyethylene glycol (PEG) hydrogel (0.1 cm). (**B**) Real image of the 20-mm long glass capillary device. (**C**,**D**) The glass capillary was inserted into the culture medium containing HepG2 or L-02 cells, and it was incubated for 24 h. Following this, cytotoxicity was measured and the control was set to 100. *n* = 4, non-significant (ns), as determined by the unpaired *t*-test.

Ethanol is readily evaporated due to its high vapor pressure. Thus, it is difficult to mimic the chronic exposure to ethanol in a cellular system. To overcome this limitation, we fabricated a device to achieve a controlled release of ethanol for a relatively long time without any addition (Figure 1A,B). The device consists of a glass capillary tube that holds 4.3 μL of ethanol. PE tubing filled with a hydrogel can temporarily prevent the evaporation of ethanol before its immersion into the media.

We checked the toxicity of the glass capillary itself to determine its biocompatibility. Empty glass capillaries were placed in the culture medium containing L-02 or HepG2 cells, which are widely used in hepatic studies. On incubating the glass capillaries with cells for 24 h, no cytotoxicity was observed (Figure 1C,D). These data indicate that the glass capillary system is biocompatible, and it can be used as a controlled release system. 

### 3.2. Glass Capillary System for the Controlled Release of Ethanol

**Figure 2:**
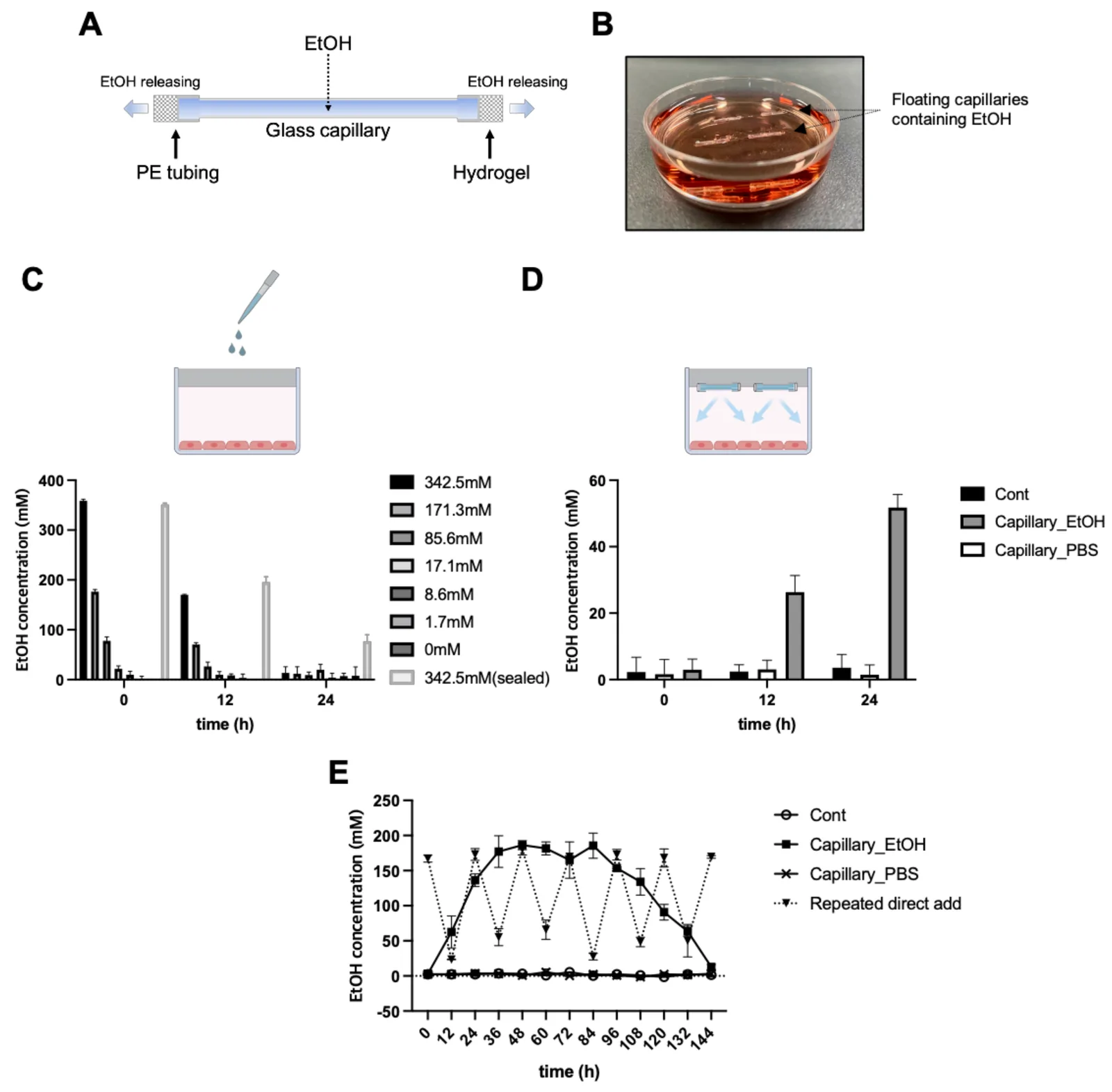
Controlled and long-term ethanol release by the capillary device. (**A**) Schematic diagram of ethanol-laden glass capillary. Ethanol is released through the PEG hydrogel (pore size: 1 nm) portions when the device is immersed in the media. (**B**) Photograph of capillaries floating on top of the culture medium. (**C**) The indicated final concentration of ethanol was directly added to 3 mL of phosphate-buffered saline (PBS) in 6-well plates. The concentration of ethanol in PBS was measured at the indicated time points. (**D**) 0 or 4 capillaries containing PBS or ethanol were immersed in 3 mL of PBS in one well of 6-well plates, and the concentration of ethanol in PBS was measured at the indicated time points. (**E**) Control, which means non-treated, or direct and repeated addition of ethanol every 24 h or 10 capillaries containing PBS or ethanol were immersed in the culture medium in 6-well plates, and the culture dishes were kept in a humidified atmosphere at 37 °C under 5% CO2. Subsequently, the concentration of ethanol in the culture medium was measured at the indicated time points. All schematics were created with BioRender.com.

To validate the capability of the device, we loaded ethanol into a capillary tube with both open ends enveloped with polyethylene tubing and half the tube containing PEG hydrogel. The pore size of PEG hydrogel is approximately 1 nm [32], enabling the controlled release of ethanol through the polymer network when immersed in the media. (Figure 2A). The ethanol-filled capillary was always floating on top of the culture medium and thus did not impact cell confluence or adhesion (Figure 2B). Subsequently, after directly adding ethanol to the PBS solution and after placing the ethanol-harboring capillary system in the PBS solution, we compared the concentration of ethanol in PBS with various ethanol concentrations. The ethanol concentrations in PBS dramatically decreased in a time-dependent manner when ethanol was directly added into the PBS solution, and the ethanol concentrations were almost zero after 24 h of ethanol addition, regardless of the ethanol concentration (Figure 2C). When the plate was sealed with parafilm, the ethanol concentration did not decrease, unlike that in the plate that was not sealed, but overall, it was confirmed that the ethanol concentration dropped rapidly. On the other hand, the capillary system containing ethanol showed a gradual increase in the ethanol concentration in PBS over time (Figure 2D). Following this, we checked the ability of the capillary apparatus to release ethanol in the cell culture system over time. Ethanol concentration in the culture medium gradually increased after the addition of capillaries, and it was maintained for 96 h. Subsequently, the ethanol concentration gradually decreased, and it reached almost zero at 144 h (Figure 2E). However, ethanol concentration in the culture medium fluctuated after direct and repeated addition of ethanol every 24 h. The direct addition of ethanol was limited to maintaining the ethanol concentration in the culture medium up to 24 h; however, the capillary system was able to maintain it for approximately 144 h. From these results, we concluded that the ethanol capillary apparatus can be a good alternative method for long-term alcohol exposure in cellular systems.

### 3.3. Long-Term Exposure of Ethanol Induces Hepatic Cytotoxicity 

**Figure 3:**
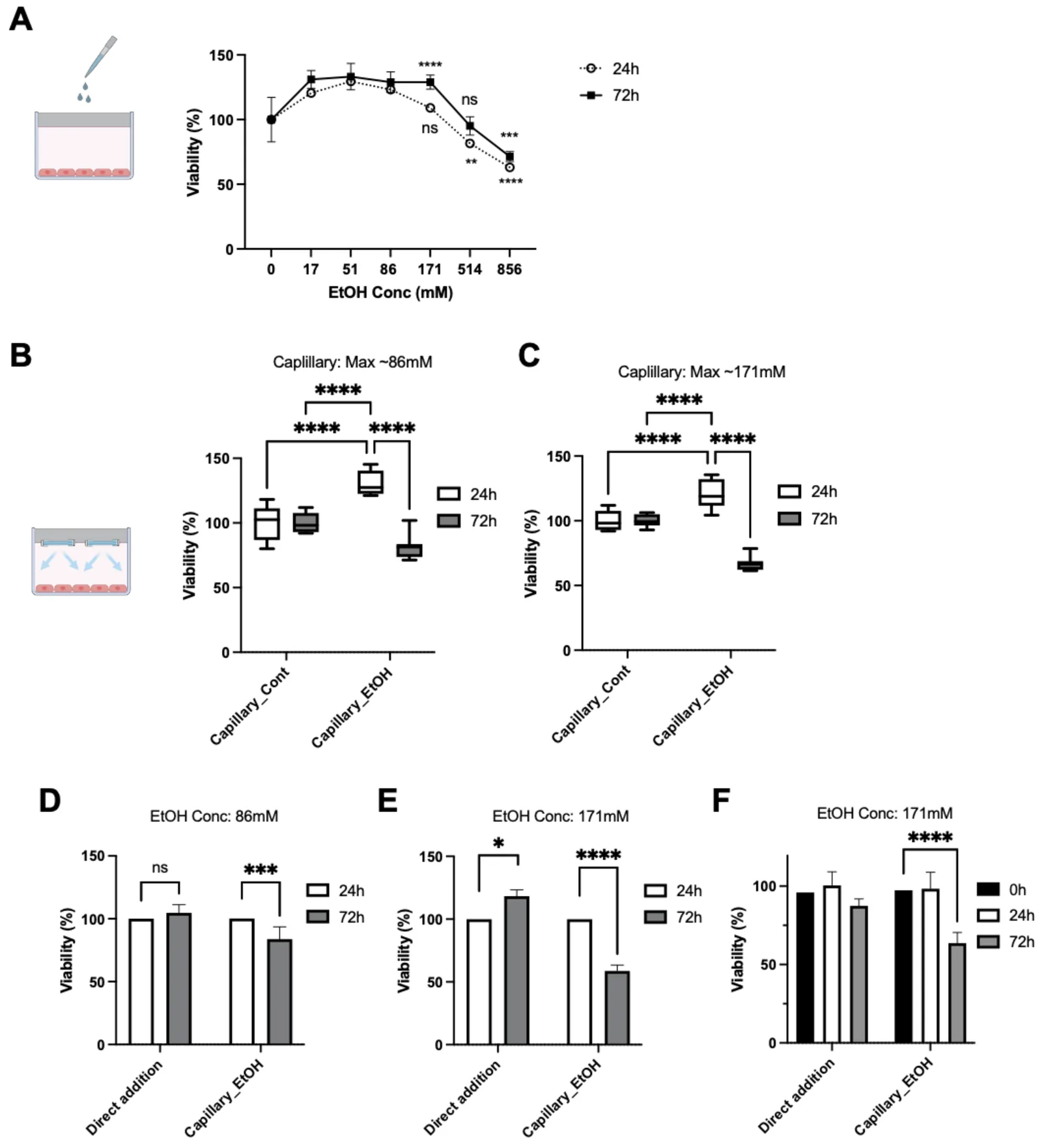
Comparison of cytotoxicities using direct addition and capillary device. (**A**) Ethanol was directly added into culture medium of HepG2 cells for the indicated concentrations. Subsequently, the cells were incubated for 24 or 72 h to measure cytotoxicity. *n* = 6, ** *p* < 0.01, *** *p* < 0.001, **** *p* < 0.0001, as determined by two-way ANOVA and Tukey’s multiple comparisons test. (**B**,**C**) Ethanol-harboring capillaries were placed in the culture medium, four capillaries containing 86 mM ethanol in 4 mL of 35 mm culture dish or 10 capillaries containing 171 mM ethanol, and this was followed by incubation for 24 or 72 h. Cells were subjected to cytotoxicity measurements, and the viability of the control capillary harboring PBS was set to 100. *n* = 8, **** *p* < 0.0001. (**D**,**E**) Ethanol was added using the direct addition or capillary system up to a concentration of 86 or 171 mM. After 24 or 72 h of incubation, the cell viability was determined. The viability of each direct addition batch was set to 100. *n* = 6, * *p* < 0.05, *** *p* < 0.001, **** *p* < 0.0001, as determined by two-way ANOVA and Tukey’s multiple comparisons test. (**F**) Ethanol was added using the direct addition or capillary system, and live and dead cells were counted at indicated time points. *n* = 4, **** *p* < 0.0001, (two-way ANOVA and Tukey’s multiple comparisons test).

We investigated the cell viabilities at various ethanol concentrations using the direct addition method. The final ethanol concentration in the culture medium from 0 mM (0%, v/v) to 171 mM (1%, v/v) did not show any cytotoxicity after 24 h of ethanol addition (Figure 3A). However, high ethanol concentrations (514 mM, 3%, or 856 mM, 5%) decreased the cell viability. Incubation with ethanol for 72 h did not dramatically change the viability of cells as compared to 24 h of incubation; rather, the viability was slightly increased but not significantly. Subsequently, we analyzed the cell viabilities of the medium containing ethanol-harboring capillaries. Capillaries that could produce ethanol concentrations of 86 or 171 mM did not show any cytotoxicity after 24 h; however, 72 h of incubation decreased the viability of cells as compared to 24 h of incubation or the capillary filled with PBS (Figure 3B,C). Compared to the cytotoxicity of direct ethanol addition and treatment with the capillary system for a final ethanol concentration of 86 mM, the capillary apparatus more significantly decreased the cell viability after 72 h of incubation than after 24 h of incubation; however, the direct ethanol treatment method did not show any significant change (Figure 3D). For 171 mM ethanol concentration, the direct ethanol addition method increased the cell viability at 72 h as compared to that at 24 h; however, the capillary system significantly decreased the viability of cells (Figure 3E,F). Therefore, the treatment of ethanol-harboring capillaries resulted in a different cell viability than the direct ethanol addition method. These data indicate that a direct addition of ethanol is suitable for acute ethanol treatment; however, for a long-term treatment, the ethanol concentration is not prolonged after 24 h. On the other hand, an ethanol capillary can maintain ethanol concentration in a culture medium for more than 72 h, and it is more suitable for the long-term exposure of cells to ethanol. 

### 3.4. Ethanol Capillary System Shows a Different Hepatic Physiology

HepG2 and L-02 hepatocytes have been shown to respond to ethanol for altered metabolism [33,34], and in many studies, HepG2 cells were treated with 100–200 mM ethanol for biological responses [35,36]. These concentrations seem to be rather high because the standard for a crackdown on drunk driving license cancellation in Korea or in the United States is approximately 0.1 to 0.2% blood alcohol concentration (equivalent to 15–30 mM). However, the lower concentration of ethanol triggered no response in other in vitro systems and our system, so we used 171 or 514 mM ethanol in this study, and we compared the concentration of remaining ethanol between the direct addition and capillary method (Figure 2C–E). It was impossible to match the ethanol concentration of the direct addition to the capillary method because of the evaporation of ethanol, so we chose the concentration which could affect cell viability: 171 or 514 mM for the direct addition; 171 mM for the capillary.

**Figure 4:**
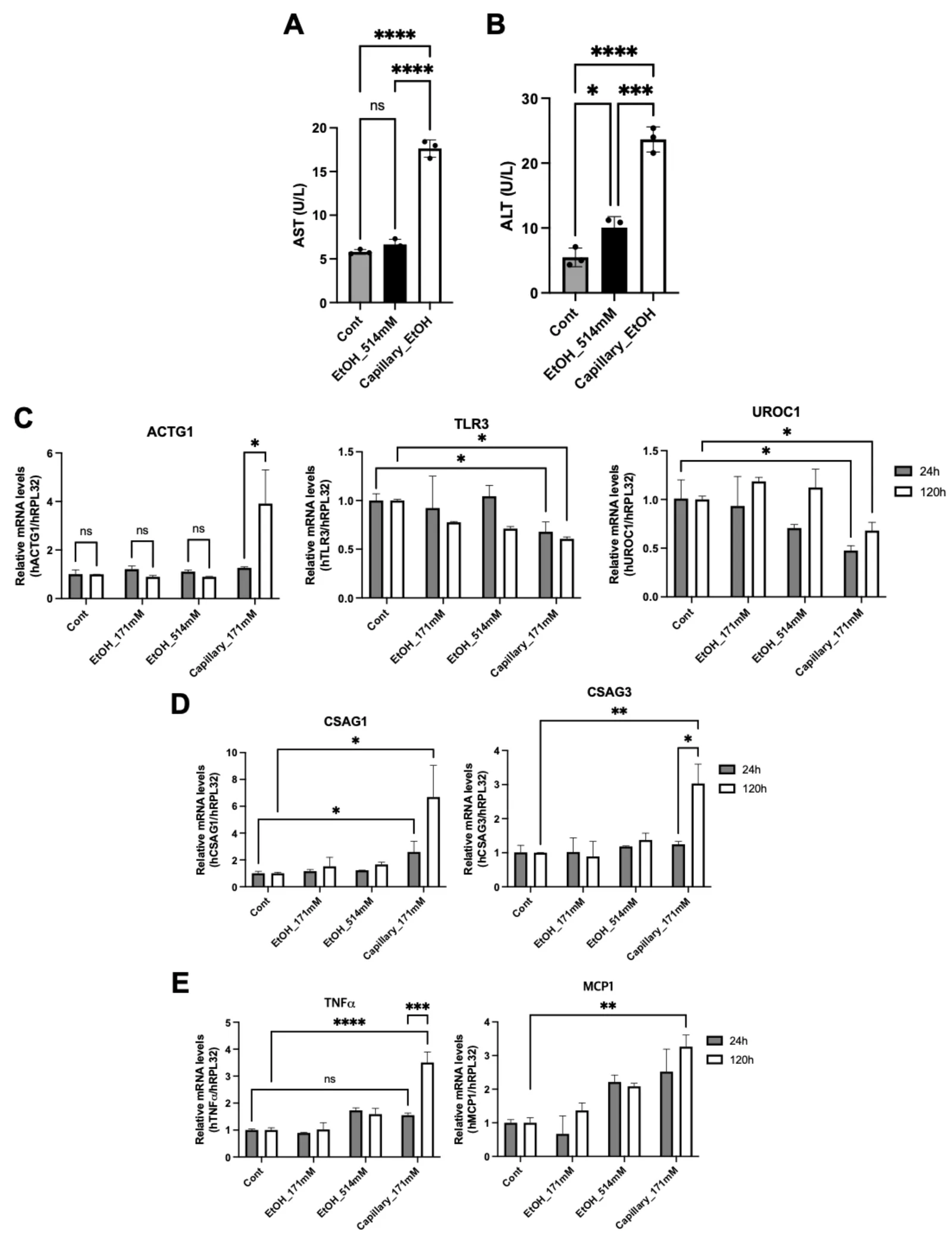
Effect of long-term exposure of ethanol to HepG2 cells. (**A**,**B**) Cells were incubated for 120 h with 514 mM ethanol using direct addition or capillaries up to 171 mM ethanol concentration. The amount of alanine transaminase and aspartate aminotransferase leakage was measured in the culture medium. *n* = 3, * *p* < 0.05, *** *p* < 0.001, **** *p* < 0.0001 (one-way ANOVA and Tukey’s multiple comparisons test). (**C**–**E**) Cells were treated with PBS for control and 171 or 514 mM ethanol by direct addition or capillaries up to 171 mM ethanol concentration, and they were incubated for 24 or 120 h. Subsequently, the cells were subjected to total RNA preparation, and the mRNA levels were quantified using the indicated gene-specific primers. The relative mRNA levels were normalized using *RPL32* mRNA levels, and the mRNA value of control batches was set to 1. *n* = 3, * *p* < 0.05, ** *p* < 0.01, *** *p* < 0.001, **** *p* < 0.0001 (two-way ANOVA and Tukey’s multiple comparisons test).

Hepatic transaminase release is a primary indicator of hepatocellular damage [37]. In this study, the ethanol-induced hepatoxicity by the direct addition of ethanol or ethanol capillaries was assessed using alanine transaminase (ALT) and aspartate aminotransferase (AST) leakage in the culture medium. After 120 h of incubation with ethanol using the indicated methods, the levels of AST and ALT increased by approximately 3–4-fold in the ethanol-harboring capillaries than in the non-ethanol-treated control; however, the direct addition of ethanol led to a smaller increase in ALT levels than the capillary system (Figure 4A,B). A direct exposure to ethanol did not significantly increase the AST levels (Figure 4A). Since direct ethanol exposure and ethanol capillary systems show different cellular physiologies, we examined the expression of several genes that were identified as key biomarkers for alcohol-related hepatocellular carcinoma [38,39]. Actin gamma 1 (*ACTG1*) mRNA levels were significantly increased by treatment with ethanol capillaries for 120 h; however, a direct exposure to ethanol did not change *ACTG1* mRNA levels (Figure 4C, left). Although 171 or 514 mM ethanol treatment for 24 h did not change the mRNA levels of toll-like receptor 3 (*TLR3*), 120 h of incubation with 514 mM ethanol slightly decreased *TLR3* expression. In contrast, the ethanol capillary system significantly decreased the expression of *TLR3* after 24 or 120 h of treatment (Figure 4C, middle). Urocanate hydratase 1 (*UROC1*) expression was significantly decreased when ethanol-harboring capillaries were treated for 24 or 120 h (Figure 4C, right). However, the direct addition of ethanol did not change the mRNA levels of *UROC1*, except when 514 mM ethanol was used for 24 h. Chondrosarcoma-associated gene 1 or 3 (*CSAG1*, *CSAG3*) also showed different expression patterns according to the direct ethanol treatment or capillary system. Incubation with ethanol capillaries for 24 or 120 h increased *CSAG1* expression levels; however, the direct ethanol addition did not cause any change (Figure 4D, left). In the case of *CSAG3*, treatment with ethanol capillaries for 120 h significantly upregulated *CSAG3* mRNA levels; however, a direct treatment with 171 or 514 mM ethanol did not show any significant change (Figure 4D, right). Tumor necrosis factor-alpha (TNFα) is a major factor in the development of alcohol-induced liver injury [40], and C–C motif chemokine ligand 2 (CCL2, also known as MCP1) is a mediator of alcohol-induced inflammatory cell activation [41]. The *TNF* and *MCP1* mRNA expression levels were also significantly increased by the treatment with ethanol-filled capillaries for 120 h (Figure 4E). These results suggest that a long-term exposure of ethanol to cells by the ethanol capillary system induces dissimilar cellular physiologies with substantially different gene expression patterns relative to acute ethanol exposure. 

### 3.5. A Long-Term Exposure to Ethanol Induces Cellular Lipid Accumulation

**Figure 5:**
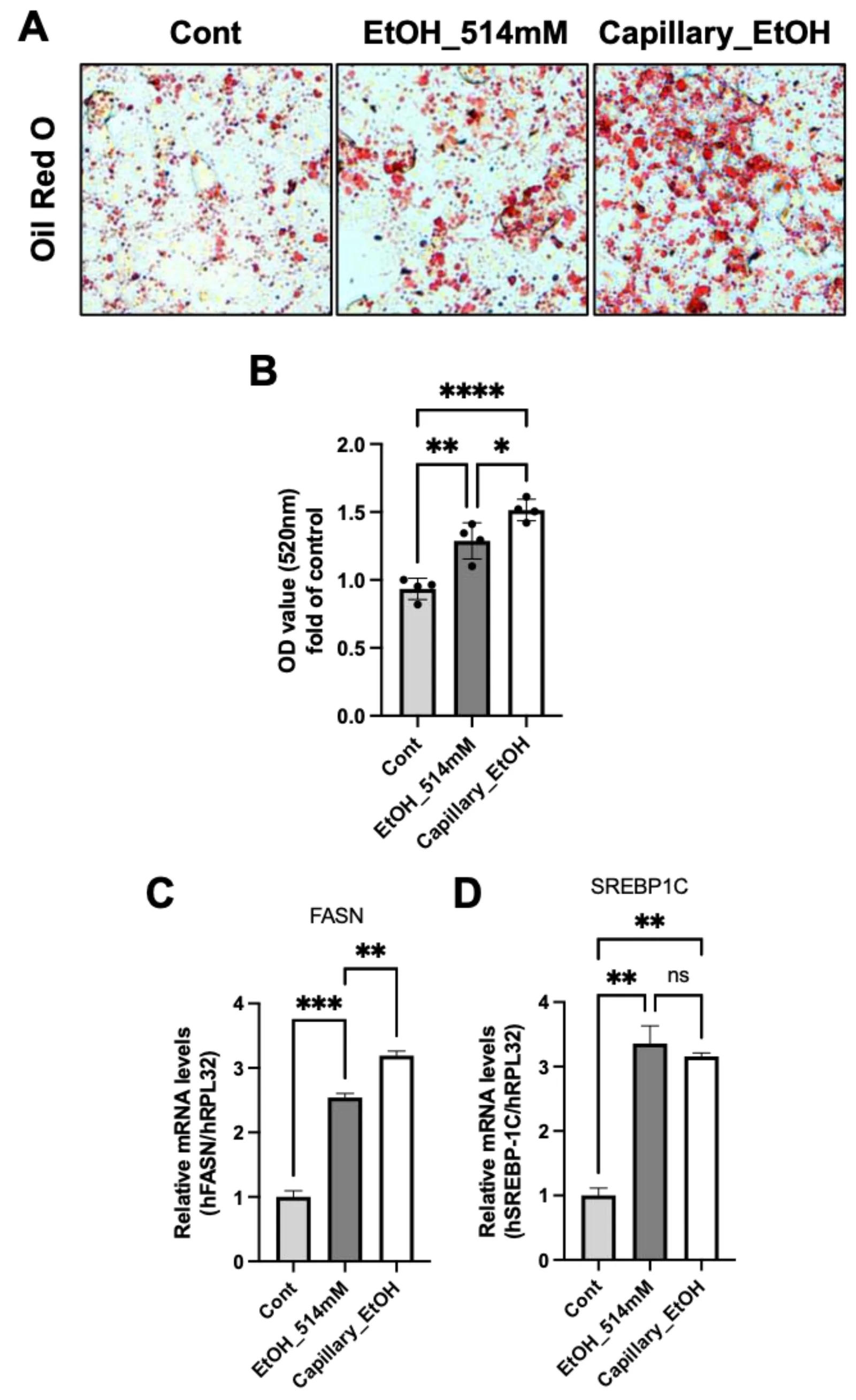
Chronic exposure of ethanol induces lipid accumulation. (**A**) HepG2 cells were treated with PBS and 514 mM ethanol using the direct addition method or ethanol-harboring capillaries up to 171 mM ethanol concentration. Subsequently, the cells were cultivated for 120 h. Lipid accumulation was determined using Oil Red O staining. (**B**) The absorbance of samples from panel A was measured at 450 nm, and the value of control was set to 1. *n* = 4, * *p* < 0.05, ** *p* < 0.01, **** *p* < 0.0001. (**C**,**D**) Control of PBS and 514 mM ethanol using direct addition or ethanol capillaries up to 171 mM ethanol concentration was added to HepG2 cells. After 120 h of incubation, the relative mRNA levels were measured using the indicated gene-specific primers, *FASN*, *SREBP1C*, and *RPL32*. Relative *FASN* or *SREBP1c* mRNA levels were quantified using endogenous *RPL32*. The relative mRNA levels of control were set to 1. *n* = 3, ** *p* < 0.01, *** *p* < 0.001.

Previous studies have demonstrated that stimulation of cells with ethanol can induce hepatic steatosis, including lipid accumulation in the cells [42,43]. In the present study, the accumulation of lipid droplets in cells was assessed through ethanol treatment using direct addition or capillaries. After 120 h of incubation with ethanol-containing capillaries, lipid accumulation was induced relative to the control treated with PBS (Figure 5A). Treatment with 171 mM ethanol in capillaries induced substantial cell death; however, we acquired images with the same levels of cell confluence for quantitative analyses. Treatment with 514 mM ethanol also accelerated lipid accumulation relative to the control, but not as much as the ethanol capillaries. For in vitro experiments, Oil Red O staining was eluted with isopropanol and quantified. As shown in Figure 5A, the ethanol-harboring capillaries induced a higher lipid accumulation than the direct ethanol addition (Figure 5B). Since the ethanol-releasing capillaries induced a high lipid accumulation in cells, the expression levels of genes involved in lipogenesis, such as fatty acid synthase (*FASN*) and sterol regulatory element binding transcription factor 1 (*SREBP-1C*), increased. Compared to the control cells, the ethanol capillary treatment caused an approximately 3.2-fold increase in the mRNA levels of *FASN* and *SREBP-1C* (Figure 5C,D). The direct addition of ethanol also increased the mRNA levels of *FASN* and *SREBP-1C*; however, *FASN* mRNA levels increased less after ethanol capillary treatment than that after capillary treatment. These results indicate that a long-term exposure to ethanol using the capillary system induces a more severe lipid accumulation than traditional ethanol addition methods. It is possible that the cytotoxic effects of the capillary ethanol shown in Figure 3 were partly due to the altered lipid metabolism. HepG2 cells have been reported to synthesize apoB-100, which is one of the essential markers of very-low-density lipoprotein, for triacylglycerol transportation to peripheral tissues [44,45,46]. Taken together, these findings suggest that the ethanol capillary system can be used as a new apparatus to mimic physiological chronic alcohol exposure conditions in the cellular system.

## 4. Discussion

Ethanol is one of the most frequently consumed organic compounds in modern society. Numerous studies have been conducted to assess the damage to human tissues and cells due to chronic ethanol intake, and the causal relationship has been well established. However, unlike in vivo studies, there are limitations in the methodology of in vitro studies involving hepatocytes. The major limitation is that it is difficult to continuously expose cells to ethanol because of ethanol volatility. Current methods are based on repeated addition of ethanol or on the saturation of the air in an incubation chamber with ethanol. In this study, we attempted to overcome the drawbacks of available treatments using an ethanol carrier with a simple design.

Consequently, we found that the sustained release of ethanol from capillaries maintained a constant plateau concentration of ethanol in the medium for at least 3 days and also confirmed that the capillaries continued releasing ethanol into the medium for up to 5 days. The in vitro results using hepatocytes showed significant increases in the levels of AST and ALT, which are biomarkers for alcohol-related hepatocellular carcinoma, and in lipid accumulation, which indicates hepatocellular damage.

In particular, it was previously reported that an increase in *ACTG1* expression and a decrease in *TLR3* expression could be used as biomarkers for the prognosis of liver cancer due to ethanol [38]. This previous report was based on the results of a study conducted on 14 patients with hepatocellular carcinoma whose underlying etiology was alcohol (GSE50579) [47]. In the present study, we also found an increase in *ACTG1* and a decrease in *TLR3* expression, which implies that the results obtained using ethanol-releasing capillaries were similar to those of in vivo analyses. Similar to our results shown in Figure 4C, a decrease in mRNA expression of *UROC1* was also observed in alcohol-induced hepatocellular carcinoma tissues, to a level of approximately one-half of that in normal tissues (*p* < 0.001).

In another recent study, certain genes were also suggested as biomarkers by analyzing tissues from patients with liver cancer that may have been caused by alcohol. *CSAG1* and *CSAG3* were representative factors for the alcohol-induced hepatocellular carcinoma [39], which implies that the results of the present study using capillaries were consistent with the in vivo findings. On the other hand, in a microarray dataset obtained in an in vitro experiment in which 75 mM ethanol was repeatedly replenished for 9 days, no statistically significant differences in the mRNA expression levels of the above genes were observed [33]. These data suggest that the exposure of hepatocytes to ethanol using the conventional ethanol exchange method may not produce physiologically meaningful outcomes.

The capillary method has the advantage of exposing cells to a certain concentration of ethanol for at least 3 days or longer and is a simple and effective method that prevents rapid changes in the ethanol concentration due to frequent medium exchange and ethanol evaporation. The limitations of this study are that the release of ethanol was not performed using media other than MEM and DMEM and that the long-term effect of the PEG hydrogel was not studied. However, PE and PEG-DA, the materials used for the capillaries in this study, are well documented to have excellent biocompatibility [48] and no reactions with specific biomolecules, and thus, there should be no significant concerns.

This study mainly focused on hepatocytes because the liver is the major organ for ethanol-caused damage. However, hepatocytes are not the only target of ethanol, and in some cases, immune cells are the major target. Malondialdehyde–acetaldehyde adducts have strong immunogenic properties, and antibodies against them are produced upon chronic exposure to ethanol [49]. The antibodies can induce autoimmune responses against hepatocytes and nonparenchymal cells [50]. The ethanol-caused disturbance of the gut–liver axis induces changes in the intestinal immune environment and might be a factor inducing alcoholic liver disease. Thus, hepatic or extrahepatic immune responses would be another proper target for the capillary ethanol release methodology [51,52].

In the present study, we assessed whether the capillary system can be used for long-term ethanol exposure experiments, and we examined several cellular physiologies and gene expression levels following treatment with ethanol capillaries. To determine the cellular and molecular changes associated with a long-term exposure of cells to ethanol, further experiments are needed to elucidate the molecular mechanism of chronic alcohol-associated diseases. Our ethanol capillary system will shed light on the methods and tools for studying the mechanism of disease development following the use of chronic alcohol at the cellular level.

## Data Availability

The original contributions presented in this study are included in the article. Further inquiries can be directed to the corresponding authors.
